# Effectiveness of a simplified cardiopulmonary resuscitation training program for the non-medical staff of a university hospital

**DOI:** 10.1186/1757-7241-22-31

**Published:** 2014-05-10

**Authors:** Tomoya Hirose, Taku Iwami, Hiroshi Ogura, Hisatake Matsumoto, Tomohiko Sakai, Kouji Yamamoto, Toshiaki Mano, Yuji Fujino, Takeshi Shimazu

**Affiliations:** 1 Department of Traumatology and Acute Critical Medicine, Osaka University Graduate School of Medicine, 2-15 Yamadaoka Suita, Osaka 565-0871, Japan; 2 Kyoto University Health Service, Yoshida Honmachi, Sakyo-ku, Kyoto 606-8501, Japan; 3 Department of Trauma, Critical Care Medicine and Burn Center, Social Insurance Chukyo Hospital, 1-1-10 Sanjo, Minami-ku, Nagoya, Aichi 457-8510, Japan; 4 Department of Medical Innovation, Osaka University Hospital, 2-15 Yamadaoka Suita, Osaka 565-0871, Japan; 5 Department of General Medicine, Osaka University Hospital, 2-15 Yamadaoka Suita, Osaka 565-0871, Japan; 6 Department of Anesthesiology and Intensive Care, Osaka University Graduate School of Medicine, 2-2 Yamadaoka Suita, Osaka 565-0871, Japan

## Abstract

**Background:**

The 2010 Consensus on Science and Treatment Recommendations Statement recommended that short video/computer self-instruction courses, with minimal or no instructor coaching, combined with hands-on practice can be considered an effective alternative to instructor-led basic life support courses. The purpose of this study was to examine the effectiveness of a simplified cardiopulmonary resuscitation (CPR) training program for non-medical staff working at a university hospital.

**Methods:**

Before and immediately after a 45-min CPR training program consisting of instruction on chest compression and automated external defibrillator (AED) use with a personal training manikin, CPR skills were automatically recorded and evaluated. Participants’ attitudes towards CPR were evaluated by a questionnaire survey.

**Results:**

From September 2011 through March 2013, 161 participants attended the program. We evaluated chest compression technique in 109 of these participants. The number of chest compressions delivered after the program *versus* that before was significantly greater (110.8 ± 13.0/min vs 94.2 ± 27.4/min, *p* < 0.0001), interruption of chest compressions was significantly shorter (0.05 ± 0.34 sec/30 sec vs 0.89 ± 3.52 sec/30 sec, *p* < 0.05), mean depth of chest compressions was significantly greater (57.6 ± 6.8 mm vs 52.2 ± 9.4 mm, *p* < 0.0001), and the proportion of incomplete chest compressions of <5 cm among all chest compressions was significantly decreased (8.9 ± 23.2% vs 38.6 ± 42.9%, *p* < 0.0001). Of the 159 participants who responded to the questionnaire survey after the program, the proportion of participants who answered ‘I can check for a response,’ ‘I can perform chest compressions,’ and ‘I can absolutely or I think I can use an AED’ increased *versus* that before the program (81.8% vs 19.5%, 77.4% vs 10.1%, 84.3% vs 23.3%, respectively).

**Conclusions:**

A 45-min simplified CPR training program on chest compression and AED use improved CPR quality and the attitude towards CPR and AED use of non-medical staff of a university hospital.

## Background

Bystander-initiated cardiopulmonary resuscitation (CPR) and the automated external defibrillator (AED) have major roles in the ‘chain of survival’ for both out-of-hospital and in-hospital cardiac arrest [1,2]. The effectiveness of the rapid response system for in-hospital cardiac arrest or critically ill patients has been reported [3,4], and in our hospital, the rapid response system was introduced in 2001. During an emergency, the rapid response team (emergency medicine doctors and nurses) are paged to rush to the stricken patient. However, education for the non-medical staff working at medical institutions, who could potentially be first responders and could activate the system, has not been established.

The 2010 Consensus on Science and Treatment Recommendations (CoSTR) Statement recommended that training should aim to ensure that learners acquire and retain the skills and knowledge that will enable them to act correctly during actual cardiac arrests, and short video/computer self-instruction courses, with minimal or no instructor coaching, combined with hands-on practice can be considered as an effective alternative to instructor-led basic life support (BLS) courses [5]. In September 2010, we introduced a 45-min simplified CPR training program consisting of instruction on chest compression and AED use with a personal training manikin for the non-medical staff working at our university hospital. From September 2011, the quality of CPR skills was recorded *via* a CPR skill report system, and a questionnaire survey on the participants’ attitudes towards CPR and AED use was conducted before and immediately after this training program. The purpose of this study was to examine the effectiveness of a simplified CPR training program for the non-medical staff working at a university hospital.

## Methods

### Study design

This was a prospective observational study that was approved by the Ethics Committee of Osaka University Graduate School of Medicine. The institutional review board waived the need for informed consent. We surveyed participants who attended this CPR training program from September 2011 through March 2013. Those eligible to participate in this program were non-medical staff working at our university hospital.

### Simplified CPR training program

**Figure 1:**
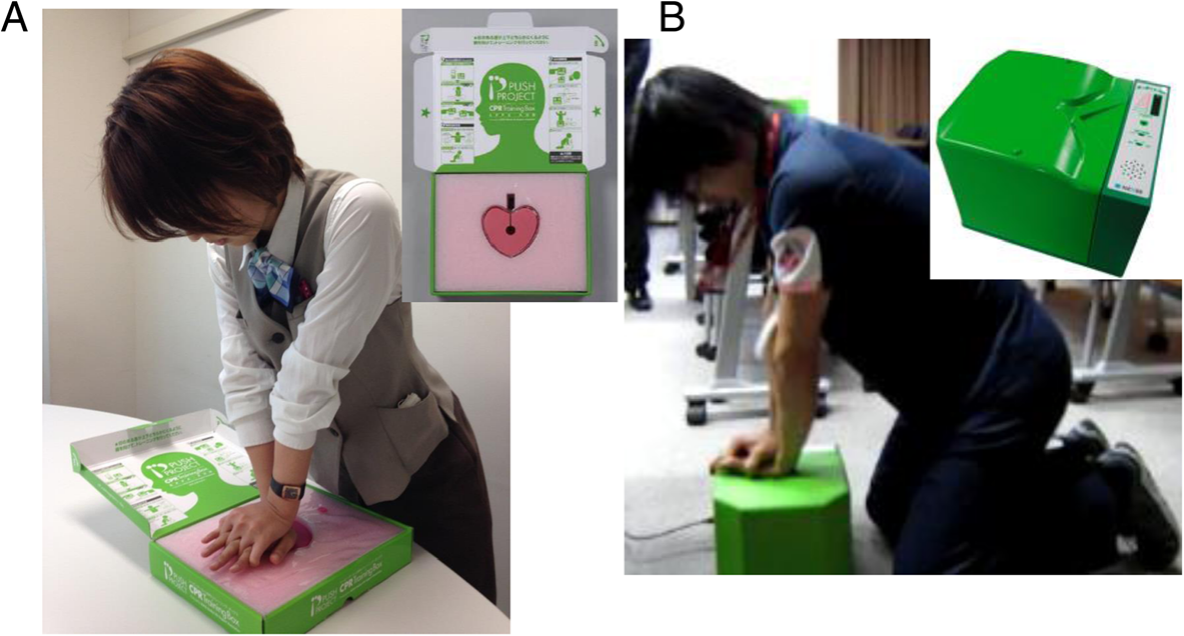
**The personal training kit. (A)** The CPR Training Box APPA-KUN®. **(B)** The CPR skill report system APPA-KUN Pro®.

**Figure 2:**
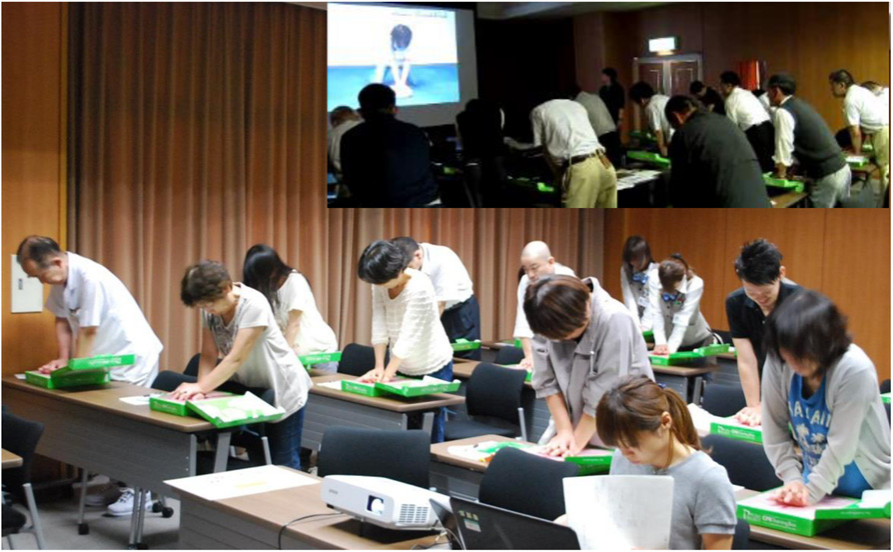
The classroom scene of the simplified cardiopulmonary resuscitation training program with personal training kit.

The simplified CPR training program consisted of instructions and practice on chest compressions and AED use with a personal training manikin, and the total time of this program was 45 minutes. We used the CPR Training Box APPA-KUN® obtained from the non-profit organisation Osaka Life Support Association, Osaka, Japan, as the personal training manikin (Figure 1A). Doctors and nurses who were instructors of the Immediate Cardiac Life Support (ICLS) course certified by the Japanese Association for Acute Medicine (JAAM) or instructors with equivalent qualifications and who were specially trained for this program instructed the participants. The instructor/participant ratio was 1:10–20. A photo of this training program in action is shown in Figure 2. The training program was DVD-based and could be held using a small number of instructors (at least one instructor was required). Table 1 shows the time schedule of this training program, which consisted of an opening speech; introduction containing a check of the participants’ knowledge about ‘check for a response’, ‘chest compressions’, and ‘AED use’; explanation of the rapid response system; simulation of an in-hospital resuscitation by DVD; practice on chest compression and AED use with the personal training manikin; and a question and answer session. We standardised the contents of the training program by using the DVD presentation.

**Table 1:** Time schedule of the simplified cardiopulmonary resuscitation training program

**Training schedule**	**Device used**	**Time (min)**
Welcome		2
Introduction (check of knowledge)	DVD	6
Rapid response system and simulation of an in-hospital resuscitation	DVD	6
Chest compression and AED use	Practice with a personal training manikin	26
Question and answer session		5
Total		45

### Evaluation of CPR skills

Before and immediately after the 45-min CPR training, CPR skills were recorded *via* the CPR skill report system APPA-KUN Pro® (Alexon, Osaka, Japan) (Figure 1B). This CPR evaluation system automatically records the number of chest compressions, interruption of chest compressions, and depth of chest compressions. The evaluation of CPR skills was performed on the participants, whose cooperation was voluntary, and the time for evaluation of CPR skills was 30 seconds due to restrictions of time and the number of CPR skill report systems available. We evaluated the participants’ CPR skill at each training program attended, as well as for all programs attended.

### Questionnaire survey evaluating participants’ attitudes towards CPR and AED use

Before and immediately after the 45-min CPR training, participants’ attitudes towards CPR and AED use were evaluated by a questionnaire survey. The question items included ‘Can you check for a response?’, ‘Can you perform chest compression?’, and ‘Can you use an AED?’. The questionnaire survey was given to all participants, and replies were anonymous. The participants provided one answer for each multiple choice question.

### Statistical analysis

All data are represented as mean ± standard deviation (SD). The Wilcoxon signed rank test was used to compare the differences between before and immediately after training, and the Wilcoxon rank sum test was used to compare the differences between the first-time and the second-time participants in the evaluation of CPR skills. A value of *p* < 0.05 was considered statistically significant. All statistical analyses were performed with JMP 9.0.2 for Windows (SAS Institute Inc., Cary, NC, USA).

## Results

### Evaluation of CPR skills

**Figure 3:**
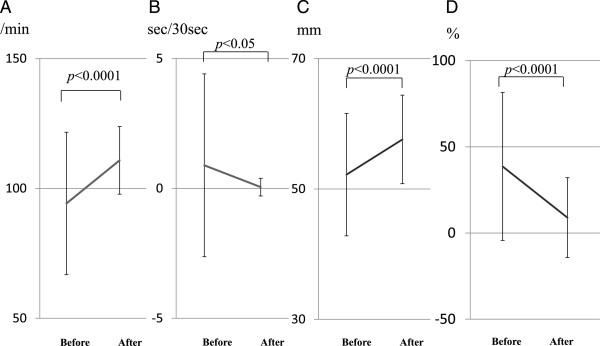
**Evaluation of cardiopulmonary resuscitation (CPR) skills for all participants before and immediately after the simplified CPR training program. (A)** Number of chest compressions, **(B)** time chest compressions were interrupted, **(C)** mean depth of chest compressions, and **(D)** proportion of incomplete chest compressions of <5 cm among all chest compressions.

From September 2011 through March 2013, 161 participants attended the program, and we evaluated the chest compression technique of 109 participants due to restrictions of time and the number of CPR skill report systems available. The study group comprised 44 men and 65 women with a mean age ± SD of 42.2 ± 14.7 years. Among the 109 participants, 57 were the first-time participants of CPR training, 48 were second-time participants, and 4 were participating for the third time or more. In the analysis of the 109 participants, the number of chest compressions was significantly greater (110.8 ± 13.0/min vs 94.2 ± 27.4/min, *p* < 0.0001) (Figure 3A), the interruption of chest compressions was significantly shorter (0.05 ± 0.34 sec/30 sec vs 0.89 ± 3.52 sec/30 sec, *p* < 0.05) (Figure 3B), the mean depth of chest compressions was significantly greater (57.6 ± 6.8 mm vs 52.2 ± 9.4 mm, *p* < 0.0001) (Figure 3C), and the proportion of incomplete chest compressions of <5 cm among all chest compressions was significantly decreased (8.9 ± 23.2% vs 38.6 ± 42.9%, *p* < 0.0001) (Figure 3D) after the program *versus* before the program.

**Figure 4:**
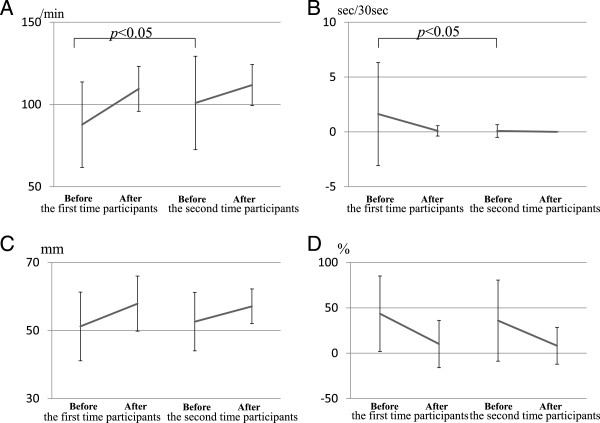
**Evaluation of cardiopulmonary resuscitation (CPR) skills according to first- and second-time attendance of the participants. (A)** Number of chest compressions, **(B)** time chest compressions were interrupted, **(C)** mean depth of chest compressions, and **(D)** proportion of incomplete chest compressions of <5 cm among all chest compressions.

We also compared CPR skill between the first and second attendance. The interval of attendance was 12.7 ± 4.3 months. Before the program, the number of chest compressions in the second-time participants was significantly greater (100.9 ± 28.4/min vs 87.7 ± 26.0/min, *p* < 0.05) (Figure 4A), and the interruption of chest compressions was significantly shorter (0.08 ± 0.58 sec/30 sec vs 1.62 ± 4.7 sec/30 sec, *p* < 0.05) (Figure 4B), compared with these values in the first-time participants. There were no significant differences between the two groups in mean depth of chest compressions (52.6 ± 8.6 mm vs 51.2 ± 10.1 mm, *p* = 0.42) (Figure 4C) and in the proportion of incomplete chest compressions of <5 cm among all chest compressions (35.9 ± 44.7% vs 43.5 ± 41.6%, *p* = 0.32) (Figure 4D). After the program as compared with before, the number of chest compressions was significantly greater (first-time participants: 109.5 ± 13.7/min vs 87.7 ± 26.0/min, *p* < 0.0001; second-time participants: 111.8 ± 12.5/min vs 100.9 ± 28.4/min, *p* < 0.05) (Figure 4A), the interruption of chest compressions was shorter (first-time participants: 0.09 ± 0.47 sec/30 sec vs 1.62 ± 4.7 sec/30 sec, *p* < 0.05; second-time participants: 0.0 ± 0.0 sec/30 sec vs 0.08 ± 0.58 sec/30 sec, *p* = 1.0) (Figure 4B), the mean depth of chest compressions was significantly greater (first-time participants: 57.9 ± 8.1 mm vs 51.2 ± 10.1 mm, *p* < 0.0001; second-time participants; 57.1 ± 5.1 mm vs 52.6 ± 8.6 mm, *p* < 0.0001) (Figure 4C), and the proportion of incomplete chest compressions of <5 cm among all chest compressions was significantly decreased (first-time participants: 10.1 ± 26.1% vs 43.5 ± 41.6%, *p* < 0.0001; second-time participants: 8.2 ± 20.3% vs 35.9 ± 44.7%, *p* < 0.0001) (Figure 4D). We excluded the participants who attended a third time or more because of the low number of participants.

### Questionnaire survey to evaluate participants’ attitudes towards CPR and AED use

Responses to the questionnaire survey were obtained from 159 (98.8%) of the 161 participants. The responder group comprised 56 men and 103 women with a mean age ± SD of 42.5 ± 14.2 years, with 95 first-time, 59 second-time, and 5 third-time or more participants.

After the program as compared with before, the proportion of participants who answered ‘I can check for a response,’ ‘I can perform chest compressions,’ and ‘I absolutely can or I think I can use an AED’ increased (81.8% vs 19.5%, 77.4% vs 10.1%, and 84.3% vs 23.3%, respectively) (Table 2).

**Table 2:** Results of the questionnaire survey to evaluate participants’ attitudes towards cardiopulmonary resuscitation and automated external defibrillator (AED) use

	**Total (N = 159)**		**First-time participants N = 95)**		**Second-time participants(N = 59)**	
	**Before**	**After**	**Before**	**After**	**Before**	**After**
Q. 1 Can you check for a response?						
I can.	31 (19.5%)	130 (81.8%)	14 (14.7%)	80 (84.2%)	15 (25.4%)	47 (79.7%)
I don’t know if I can.	103 (64.8%)	29 (18.2%)	58 (61.1%)	15 (15.8%)	42 (71.2%)	12 (20.3%)
I can’t.	21 (13.2%)	0 (0%)	20 (21.1%)	0 (0%)	1 (1.7%)	0 (0%)
I shouldn’t. More skillful people should.	4 (2.5%)	0 (0%)	3 (3.2%)	0 (0%)	1 (1.7%)	0 (0%)
Q. 2 Can you perform chest compression?						
I can.	16 (10.1%)	123 (77.4%)	8 (8.4%)	75 (78.9%)	7 (11.8%)	46 (78.0%)
I don’t know if I can.	88 (55.3%)	36 (22.6%)	38 (40.0%)	20 (21.1%)	46 (78.0%)	13 (22.0%)
I can’t.	49 (30.8%)	0 (0%)	43 (45.3%)	0 (0%)	6 (10.2%)	0 (0%)
I shouldn’t. More skillful people should.	6 (3.8%)	0 (0%)	6 (6.3%)	0 (0%)	0 (0%)	0 (0%)
Q. 3 Can you use an AED?						
I absolutely can.	4 (2.5%)	27 (17.0%)	2 (2.1%)	17 (17.9%)	1 (1.7%)	9 (15.3%)
I think I can.	33 (20.8%)	107 (67.3%)	14 (14.7%)	67 (70.5%)	18 (30.5%)	38 (64.4%)
I don’t know if I can.	58 (36.5%)	19 (11.9%)	31 (32.6%)	7 (7.4%)	25 (42.4%)	10 (16.9%)
I think I can’t.	47 (29.6%)	4 (2.5%)	34 (35.8%)	3 (3.2%)	12 (20.3%)	1 (1.7%)
I absolutely can’t.	17 (10.7%)	2 (1.3%)	14 (14.7%)	1 (1.1%)	3 (5.1%)	1 (1.7%)

In the second-time participants, the proportion of participants who answered ‘I can’t or shouldn’t check for a response’ was smaller (2 [3.4%] vs 23 [24.2%]), who answered ‘I can’t or shouldn’t perform chest compression’ was smaller (6 [10.2%] vs 49 [51.6%]), and who answered ‘I absolutely can’t or I think I can’t use an AED’ was smaller (15 [25.4%] vs 48 [50.5%]) than that of the first-time participants. After the program as compared with before, the proportion of participants who answered ‘I can check for a response’ was greater (first-time participants: 80 [84.2%] vs 14 [14.7%]; second-time participants: 47 [79.7%] vs 15 [25.4%]), who answered ‘I can perform chest compressions’ was greater (first-time participants: 75 [78.9%] vs 8 [8.4%]; second-time participants: 46 [78.0%] vs 7 (11.8%]), and who answered ‘I absolutely can or I think I can use an AED’ was greater (first-time participants: 84 [88.4%] vs 16 [16.8%]; second-time participants: 47 [79.7%] vs 19 [32.2%]) (Table 2).

## Discussion

In this study, we showed the effectiveness of a simplified 45-min CPR training program for non-medical staff working at a university hospital that improved both the quality of CPR and the attitude of the staff towards CPR and AED use.

CPR and AED use by bystanders are very important in the ‘chain of survival’ for both out-of-hospital and in-hospital cardiac arrests to improve patient survival [1,2]. It was reported that survival to hospital discharge is still about 15% to 20% after in-hospital-cardiac arrest [6]. The non-medical staff working at a large hospital potentially can be the first responders for patients requiring CPR in most settings. To improve the ‘chain of survival’, especially in the first three links of the chain and to quickly activate the rapid response team, an adequate educational program is needed for these personnel.

Recently, animal and clinical research suggested that bystander-initiated cardiac-only resuscitation is at least as effective as conventional CPR for ventricular fibrillation (VF) or short periods of untreated arrest [7]–[10]. In addition, it was reported that cardiac-only resuscitation without mouth-to-mouth ventilation was easier to learn and perform and made it possible for the general public to perform a greater number of appropriate chest compressions than with the conventional CPR program [11,12]. Therefore, we introduced a 45-min simplified CPR training program consisting of instruction and practice in chest compression and AED use with a personal training manikin for the non-medical staff working at our university hospital because we needed to educate a number of these personnel in a short time.

In this study, we successfully demonstrated an improvement in the quality of CPR after the simplified CPR training course (Figure 3). The 2010 CoSTR Statement emphasised the need for improving the quality of CPR to increase patient survival after cardiac arrest [5]. Christenson et al. reported that the chest compression fraction appears to be an important determinant of survival from cardiac arrest [13]. It was also reported that shallower chest compressions correlated significantly with a decrease in successful defibrillation [14,15]. Now, the rescuer should give chest compressions to a depth of at least 5 cm and at a rate of at least 100 times per minute, allow full chest recoil after each compression, and minimise interruptions in chest compression [5]. The improvement in the quality of CPR seen in the participants after the simplified CPR training may lead to an improvement in the prognosis of patient suffering in-hospital cardiac arrest in our hospital. Further analysis of serial in-hospital cardiac arrest statistics would prove this hypothesis.

In this study, we conducted a questionnaire survey to evaluate participants’ attitudes towards CPR and AED use and demonstrated an improvement in their attitudes after the training (Table 2). Our result was consistent with that of a previous report that indicated that in an actual emergency setting, the participants of CPR training were more likely to perform CPR than those without the experience of CPR training [16]. However, Dwyer reported that even if the participants answered that they were confident that they could initiate CPR after CPR training, they could not perform CPR adequately in an actual emergency situation [17]. Therefore, further research as to whether the result in this study will lead to an increase in the initiation of CPR in an actual emergency situation is needed.

We found that the quality of CPR and the participants’ attitude towards CPR and AED use were better in the second-time participants than in the first-time participants before CPR training (Figure 4, Table 2). BLS and advanced cardiac life support (ACLS) knowledge and skills can deteriorate in as little as 3 to six months [5,18,19]. Therefore, more frequent assessments or refresher training is recommended to maintain knowledge and skills [5]. In the present study, the interval of attendance from the last CPR training was 12.7 ± 4.3 months, and even though the CPR skills and attitude towards CPR of the second-time participants were better than those without experience of previous CPR training, they were not still sufficient (Figure 4D). As such, repeated attendance at an interval of less than 1 year would be desirable. The optimal interval between CPR training programs to maintain the quality of CPR and attitude towards CPR and AED use requires further clarification.

The 45-min time of this training program was shorter than that of conventional BLS or ACLS courses, and this training could be conducted by at least one instructor. Therefore, less burdens on time and expense exist for either the participants or the instructors. Because our program improved the quality of CPR and attitude towards CPR and AED use of the participants, this program could be considered not only in medical but also in non-medical institutions when implementing CPR and AED use.

As limitations of the present study, first, due to restrictions of time and the number of available CPR skill report systems, the evaluation of CPR skills was performed for only 30 seconds on 109 participants in whom cooperation was voluntary. Second, we did not test the practical skill of the participants on the AED due to restrictions of time. Further study is needed to evaluate the appropriate use of an AED after the training program. Third, there is no data on longer-term retention of skills. Forth, whether the participants of this CPR training could actually perform CPR in an emergency situation is unknown.

## Conclusion

A simplified 45-min CPR training program combining instruction and practice in chest compression and AED use improved the quality of CPR and the attitude towards CPR and AED use of the non-medical staff working at a university hospital. Further study to reveal the optimal interval for conducting the CPR training program to maintain the quality of CPR and a positive attitude towards CPR and AED use is needed.

## Competing interests

The authors have no conflicts of interest to declare in relation to this manuscript.

## Authors’ contributions

TH, TI, and TS designed the study. TH, HM, TS, and KY collected and generated the data. TH wrote the first draft. TH, TI, HO, KY, TM, YF and TS analyzed the data and helped to draft the manuscript. All of the authors read and approved the final manuscript.
